# Gallstone Ileus Causing Mechanical Bowel Obstruction in a Patient With Caroli's Disease: A Case Report

**DOI:** 10.7759/cureus.93125

**Published:** 2025-09-24

**Authors:** Inês Soares, Ana Sofia Silva, Mariana Batista, Tiago Fernandes

**Affiliations:** 1 Internal Medicine, Unidade Local de Saúde de Gaia e Espinho, Vila Nova de Gaia, PRT

**Keywords:** abdominal pain, caroli’s disease, gallstone ileus, hepatojejunostomy, liver

## Abstract

Gallstone ileus is a rare but significant cause of abdominal pain and mechanical bowel obstruction. Its management usually involves minimally invasive surgical intervention.

This case report describes a 65-year-old woman with a history of Caroli disease and prior abdominal surgery, who presented with a one-month history of progressive, cramp-like abdominal pain, accompanied by nausea and intermittent vomiting. Due to these persistent symptoms, she underwent a CT scan, which confirmed gallstone ileus with obstruction of a jejunal loop, necessitating surgical removal of the gallstone.

This case underscores the importance of integrating a patient’s medical history with their presenting symptoms and maintaining a high level of clinical suspicion for rare but serious causes of mechanical bowel obstruction that may otherwise be overlooked.

## Introduction

Caroli disease is a rare congenital disorder characterized by abnormal development of the intrahepatic bile ducts, leading to ductal dilation and cyst formation. Its estimated prevalence is approximately 1 in 1,000,000 individuals, making it an exceptionally uncommon condition [1]. This disorder is associated with several clinically significant complications. Recurrent cholangitis occurs in up to two-thirds of patients, typically presenting with fever, jaundice, and abdominal pain. Hepatolithiasis, or stone formation within the dilated bile ducts, affects roughly 30% of patients and can result in obstruction and infection. Less frequent but serious complications include biliary cirrhosis and cholangiocarcinoma, with reported rates ranging from 2.7% to 37.5% and an overall incidence of 6.6%, highlighting the potential long-term morbidity associated with the disease [2,3]. Management often involves both medical and surgical strategies. Localized disease may be treated with surgical resection of the affected liver segment, which can be curative, whereas advanced cases often require liver transplantation [3].

Gallstone ileus, although rare, is a severe complication of cholelithiasis that occurs when a gallstone enters and obstructs the gastrointestinal tract. It is more common in older adults, particularly women, due to the higher prevalence of gallstones in this population. Additional risk factors include a history of recurrent cholecystitis, formation of a biliary-enteric fistula, and prolonged biliary inflammation. Gallstone ileus accounts for 1-4% of mechanical bowel obstructions and poses diagnostic challenges due to its nonspecific symptoms, such as abdominal pain, nausea, and vomiting. Diagnosis is typically made through abdominal computed tomography (CT), and treatment involves surgical removal of the impacted gallstone and resolution of the obstruction [1,4].

The simultaneous occurrence of Caroli disease and gallstone ileus is extremely rare, reflecting the distinct pathophysiology of the two conditions: Caroli disease primarily involves intrahepatic bile duct dilation, whereas gallstone ileus results from gallstone migration through a biliary-enteric fistula into the gastrointestinal tract [5,6]. To date, gallstone ileus in the context of Caroli disease has only been reported in isolated case reports [7]. Documenting such cases is clinically important, as it raises awareness of rare but serious complications in patients with complex biliary and gastrointestinal histories, thereby facilitating timely diagnosis and management.

## Case presentation

A 65-year-old woman presented with a one-month history of cramp-like abdominal pain and post-prandial vomiting. She denied constipation. She had a medical history of Caroli's Disease (Todani’s Classification type V) and was on ursodeoxycholic acid (UDCA) (15mg/kg/dia). Because of this condition, she had previously undergone a cholecystectomy and left hepatectomy in 1983, as well as a resection of the 7th and 8th liver segments in 1986. In the first surgery, a biliary-enteric anastomosis was created to prevent recurrent obstruction of the intrahepatic bile ducts [8]. Upon examination at the emergency department, an abdominal ultrasound was performed, which revealed a nodular formation with a diameter of 4.2 cm at the biliodigestive anastomosis. No abnormal blood results were found.

**Figure 1:**
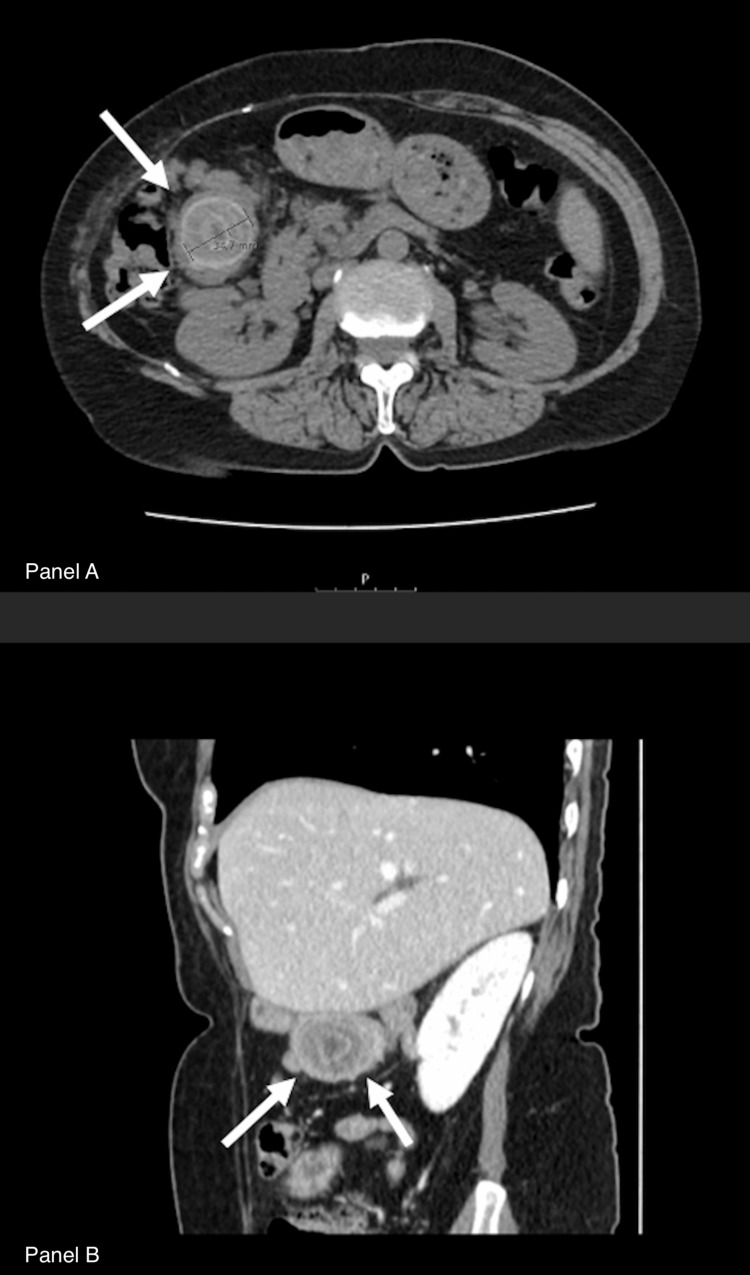
Abdominal CT scan showing the gallstone impacted in the small bowel Panel A: Transverse plane. Arrows showing the biliary stone. Panel B: Coronal plane. Arrows showing the biliary stone.

To confirm the nature of the nodular formation, a CT scan was performed two months after, which revealed that the nodular image, with a similar size, had migrated from the biliodigestive anastomosis to the jejune, obstructing a jejunal loop (Figure 1). 

**Figure 2:**
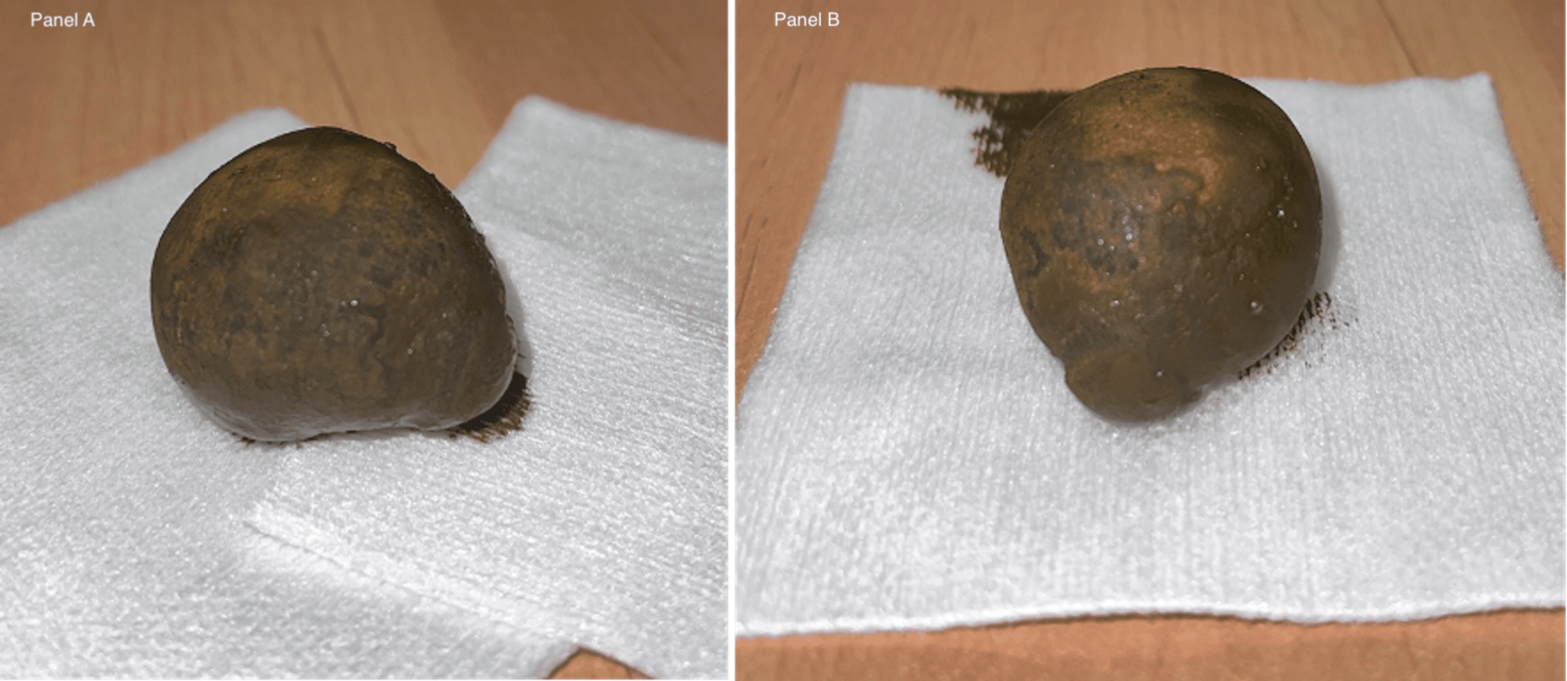
Photo of the gallstone removed Panels A and B show different views of the gallstone.

The patient underwent laparoscopic enterolithotomy for recurrent abdominal pain and anorexia. The obstructing gallstone was removed through a small intestinal incision, the bowel was inspected for additional stones, and the procedure was completed without complications. A gallstone was successfully removed during the procedure (Figure 2). During the postoperative course, the patient experienced an uncomplicated recovery, with no surgical or medical complications, and was discharged three days after surgery.

## Discussion

This case highlights a rare presentation of gallstone ileus as a complication of Caroli’s disease, emphasizing the interplay between congenital biliary abnormalities, prior surgical interventions, and mechanical bowel obstruction. The patient had multiple risk factors predisposing her to gallstone ileus, including advanced age, female sex, a history of cholelithiasis, prior cholecystectomy, and, most notably, the presence of a biliary-enteric anastomosis [5, 6, 9, 10]. The latter, created to prevent recurrent intrahepatic bile duct obstruction, inadvertently facilitated the passage of a large biliary calculus into the gastrointestinal tract, ultimately leading to small bowel obstruction. 

UDCA, commonly used in Caroli’s disease to reduce bile stasis and prevent lithogenesis, has demonstrated some efficacy in dissolving cholesterol-based stones [11-13]. However, despite chronic UDCA therapy, stone formation persisted, leading to complications. This suggests that while UDCA may help mitigate small stone formation, it does not entirely eliminate the risk of biliary calculi, particularly in patients with altered bile flow dynamics due to prior surgical modifications [7].

Diagnosing gallstone ileus can be challenging due to its nonspecific symptoms, as demonstrated by the patient’s one-month history of cramp-like abdominal pain and postprandial vomiting. The initial ultrasound revealed a nodular formation at the site of the anastomosis, raising concerns about possible anastomotic complications or neoplastic recurrence. The definitive diagnosis was made two months later when a CT scan confirmed the migration of the formation into the jejunum, leading to obstruction. This highlights the importance of serial imaging in patients with complex biliary histories. Although Rigler’s triad (pneumobilia, small bowel obstruction, and an ectopic gallstone) is a well-known radiologic hallmark,​​​​ [7] this case did not meet all three criteria, likely due to altered anatomy from the biliary-enteric anastomosis. 

Surgical intervention remains the definitive treatment for gallstone ileus. In this case, laparoscopic enterolithotomy was successfully performed, offering a minimally invasive approach with reduced morbidity and faster recovery. The patient’s uneventful postoperative course and discharge on day three underscore the safety and efficacy of this technique in select patients [14]. Alternative strategies, such as endoscopic stone retrieval or staged percutaneous approaches, may be considered in high-risk patients, while recurrent hepatolithiasis or extensive biliary disease may necessitate segmental hepatectomy or liver transplantation [15-17].

This case also emphasizes the importance of long-term follow-up in patients with Caroli disease, particularly those with biliary-enteric communications. Periodic imaging and laboratory monitoring are essential to detect recurrent stones, biliary obstruction, cholangitis, or rare complications such as cholangiocarcinoma.

By documenting this rare presentation, the case contributes to existing knowledge of Caroli disease complications, reinforces the role of biliary-enteric anatomy in stone formation, and highlights the importance of individualized management and vigilant surveillance.

## Conclusions

This case highlights the extreme rarity of gallstone ileus occurring in a patient with Caroli disease, a condition characterized by segmental dilation of the intrahepatic bile ducts. The dilated ducts and associated biliary-enteric communication likely promoted bile stasis and bacterial colonization, facilitating the formation of biliary stones that ultimately migrated into the gastrointestinal tract. The patient underwent laparoscopic enterolithotomy and experienced an uncomplicated postoperative course, being discharged three days after surgery.

The coexistence of these two conditions has only been reported in isolated cases, making this report clinically valuable. It underscores the need for long-term follow-up of patients with Caroli disease, particularly those with biliary-enteric communications, as they remain at risk for recurrent stone formation and other biliary complications. Early recognition, prompt imaging, and timely surgical intervention are crucial for achieving favorable outcomes. Reporting such rare cases contributes to the understanding of the mechanisms behind gallstone formation in Caroli disease and guides clinicians in managing similarly complex presentations.
